# Process evaluation of a reablement training program for homecare staff to encourage independence in community-dwelling older adults

**DOI:** 10.1186/s12877-020-01936-7

**Published:** 2021-01-06

**Authors:** Teuni H. Rooijackers, G. A. Rixt Zijlstra, Erik van Rossum, Ruth G. M. Vogel, Marja Y. Veenstra, Gertrudis I. J. M. Kempen, Silke F. Metzelthin

**Affiliations:** 1 Department of Health Services Research, Care and Public Health Research Institute, Maastricht University, P.O. Box 616, 6200 MD Maastricht, The Netherlands; 2 Living Lab in Ageing and Long-Term Care, Maastricht, The Netherlands; 3 Zuyd University of Applied Sciences, Research Center for Community Care, Academy of Nursing, P.O. Box 550, 6400 AN Heerlen, The Netherlands; 4 Burgerkracht Limburg, P.O. Box 5185, 6130 PD Sittard, The Netherlands

**Keywords:** Activities of daily living, Behavior and behavior mechanisms, Home and community based care and services, Independence, Process evaluation, Reablement, Self-care, Training program

## Abstract

**Background:**

Many community-dwelling older adults experience limitations in (instrumental) activities of daily living, resulting in the need for homecare services. Whereas services should ideally aim at maintaining independence, homecare staff often take over activities, thereby undermining older adults’ self-care skills and jeopardizing their ability to continue living at home. Reablement is an innovative care approach aimed at optimizing independence. The reablement training program ‘Stay Active at Home’ for homecare staff was designed to support the implementation of reablement in the delivery of homecare services. This study evaluated the implementation, mechanisms of impact and context of the program.

**Methods:**

We conducted a process evaluation alongside a 12-month cluster randomized controlled trial, using an embedded mixed-methods design. One hundred fifty-four homecare staff members (23 nurses, 34 nurse assistants, 8 nurse aides and 89 domestic workers) from five working areas received the program. Data on the implementation (reach, dose, fidelity, adaptations and acceptability), possible mechanisms of impact (homecare staff's knowledge, attitude, skills and support) and context were collected using logbooks, registration forms, checklists, log data and focus group interviews with homecare staff (*n* = 23) and program trainers (*n* = 4).

**Results:**

The program was largely implemented as intended. Homecare staff's average compliance to the program meetings was 73.4%; staff members accepted the program, and particularly valued its practical elements and team approach. They experienced positive changes in their knowledge, attitude and skills about reablement, and perceived social and organizational support from colleagues and team managers to implement reablement. However, the extent to which homecare staff implemented reablement in practice, varied. Perceived facilitators included digital care plans, the organization’s lump sum funding and newly referred clients. Perceived barriers included resistance to change from clients or their social network, complex care situations, time pressure and staff shortages.

**Conclusions:**

The program was feasible to implement in the Dutch homecare setting, and was perceived as useful in daily practice. Nevertheless, integrating reablement into homecare staff's working practices remained challenging due to various personal and contextual factors. Future implementation of the program may benefit from minor program adaptations and a more stimulating work environment.

**Trial registration:**

ClinicalTrials.gov (Identifier NCT03293303). Registered 26 September 2017.

**Supplementary Information:**

The online version contains supplementary material available at 10.1186/s12877-020-01936-7.

## Background

Due to the ageing of the population, many high-income countries nowadays stimulate an ‘ageing-in-place’ policy to enable older adults to live independently at home for as long as possible [1, 2]. Consequently, the vast majority of older adults remain living at home, which is in line with their stated preferences [3]. However, many older adults suffer from limitations in (instrumental) activities of daily living ((I)ADLs), which may result in the need for homecare services [4]. In the Netherlands, these services are provided by different types of homecare workers. A team of nurses, nurse assistants and nurse aides provide personal and nursing care, often through short visits to clients several times a week. Domestic workers provide domestic support; they usually visit clients once per week for a couple of hours. Whereas homecare staff should ideally aim at maintaining older adults’ independence, they often take over (I)ADLs, as they are used to ‘doing for’ rather than ‘doing with’ older adults [5]. This practice may undermine older adults’ self-care skills and jeopardize their ability to continue living at home [6].

Innovative care approaches may support homecare staff in implementing the ‘doing with’ approach in daily homecare practice. This fits well with the holistic and person-centered philosophy of reablement [7]. Reablement, also termed restorative care, aims to enhance an individual’s (physical) functioning, increase or maintain their independence in meaningful activities of daily living, and reduce their need for long-term services [7–9]. The approach includes an initial comprehensive needs assessment followed by regular reassessments and the development of a goal-oriented support plan. A trained and coordinated interdisciplinary team supports the individual to achieve their goals, if applicable, through participation in daily activities, home modifications, assistive devices, and involvement of the social network. Previous studies have indicated that the knowledge, willingness and skills of healthcare staff to adopt a ‘reabling’ approach are essential to the success of reablement [7, 9, 10]. In addition, contextual factors, such as older adults’ receptiveness to trying new things are considered vital [7, 10]. Despite these insights, little is currently known about the implementation process of reablement, its underlying theory and mechanisms of impact, and the influence of contextual factors [11–14]. A more profound understanding of what reablement entails in terms of staff training and practice delivery may shed light on this.

The Dutch reablement training program ‘Stay Active at Home’ was recently designed to integrate reablement in the delivery of homecare services [15, 16]. The program seeks to equip homecare staff (nurses, nurse assistants, nurse aides and domestic workers) with knowledge, attitude, skills, social and organizational support to implement reablement in daily homecare practice. With this, we aim to change homecare staff's behavior from taking over the activities of older adults towards encouraging older adults to perform activities for themselves, thereby supporting older adults in managing their everyday their everyday lives as independently as possible [6, 17]. We pre-tested the program in a pilot study and an exploratory trial to obtain insight into staff's experiences with the program [15, 18]. They perceived the program as useful to implement reablement, but required more support in mastering particular skills, such as conversational and goal-setting skills, and in dealing with challenging situations. Further research on the program in terms of a process evaluation may provide a more detailed understanding of the program’s functioning. The Medical Research Council (MRC) framework for designing and evaluating complex interventions recommends conducting a process evaluation that assesses the implementation process, clarifies causal mechanisms and identifies contextual factors [19]. Therefore, the aim of this paper was to evaluate the implementation, mechanisms of impact and context the reablement training program 'Stay Active at Home' in the homecare setting.

## Methods

### Study design

This process evaluation was conducted alongside a 12-month cluster randomized controlled trial (cRCT) in the Dutch homecare setting. For logistical reasons, the program’s implementation and evaluation occurred in two waves; the first wave started in October 2017, the second in January 2018. We used an embedded mixed-methods design in which quantitative data were embedded within a mainly qualitative methodology [20], thereby adhering to components of the Consolidated Criteria for Reporting Qualitative Research, the Good Reporting of a Mixed Methods Study checklist, and the Consolidated Standards of Reporting Trials statement (extension for cluster trials) [21–23]. Details of the design were previously reported [16] and the study is registered at ClinicalTrials.gov (Identifier NCT03293303).

### Setting

The healthcare organization involved has divided its region into seven working areas that are sub-divided into small-scale self-directed nursing teams, with on average 11 teams per working area (range 3–28). Each team consists of baccalaureate-educated registered nurses, vocationally-trained registered nurses, (certified) nurse assistants and nurse aides. The team is jointly responsible for providing personal care (e.g., washing and dressing); nursing care (e.g., medication management) is provided by registered nurses only. One of the nurses on the team, the district nurse, has a more supervising and coordinating role. In addition, each working area includes a group of domestic workers who provide domestic support (e.g., vacuuming and doing the laundry). They are not registered and do not need a formal domestic qualification. In this article, we used the term ‘nursing team members’ when referring to nurses, nurse assistants and nurse aides; the term ‘homecare staff’ was used when referring to nursing team members and domestic workers simultaneously.

### Participants

The healthcare organization appointed ten nursing teams from five working areas (two teams per area), which were pre-stratified based on area and randomized into the intervention or control group, together with their clients and, if applicable, clients’ domestic workers. The current article focused on homecare staff in the intervention group, as they directly engaged in the program. Nursing team members were eligible to participate in the program if they worked in one of the intervention group nursing teams at the start of the study [16]. Domestic workers were eligible if they provided services to clients of one of the intervention groups nursing teams at the start of the study. Since the provision of the program was considered as a regular quality improvement strategy, all eligible staff members who were enrolled to the intervention group were expected to participate in the program. In addition, program trainers were included: two employees of the healthcare organization with extensive experience in training care staff and a background in homecare management and education, and two researchers with expertise in the program content and a background in occupational therapy (author SFM), and public health (author THR).

### Intervention

**Fig. 1:**
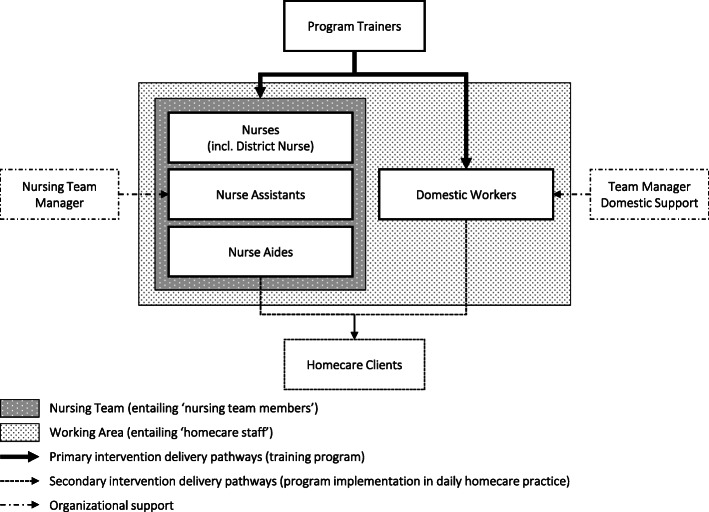
Implementation process of the ‘Stay Active at Home’ program for homecare staff

The ‘Stay Active at Home’ reablement training program aims to improve the independence of homecare clients (*secondary intervention delivery pathways*) through equipping homecare staff with knowledge, attitude, skills and social and organizational support from colleagues and team managers to implement reablement in daily homecare practice (*primary intervention delivery pathways*). The program’s implementation process is outlined in Fig. 1.

**Fig. 2:**
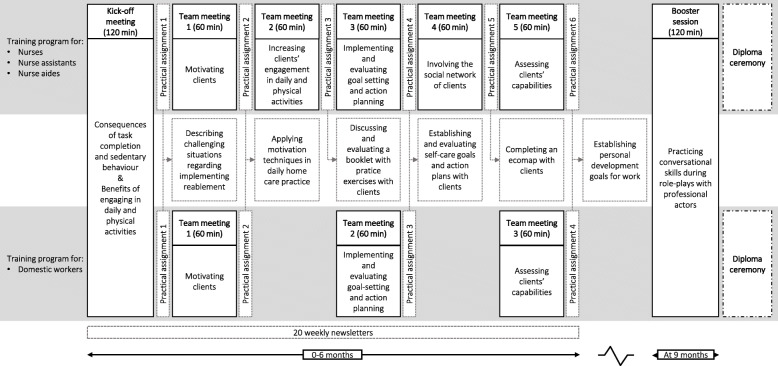
Format and content of the ‘Stay Active at Home’ program for homecare staff

The program consisted of program meetings, practical assignments and periodic newsletters. Program meetings were divided into a kick-off meeting (120 min), bi-(monthly) team meetings (60 min each) over a 6-month period, and a booster session at 9 months (120 min). Although meetings were largely similar for all staff members, distinct trajectories were designed for nursing team members and for domestic workers. The joint kick-off meeting for all staff members of the same working area provided background information on why a re-orientation of homecare was needed. Each team meeting then addressed a skill to facilitate the implementation of reablement in practice: 1) motivating clients, 2) increasing clients’ engagement in daily and physical activities, 3) implementing goal setting and action planning, 4) involving the social network of clients, and 5) assessing clients’ capabilities. Domestic workers received fewer meetings than nursing team members due to a lower annual time-budget for training activities in the Netherlands. Practical assignments were distributed at the end of each meeting to practice skills in-between the meetings. As part of the assignments, nursing team members also received a booklet with practice exercises and an ecomap (i.e. diagram depicting relationships between a client and his/her social network). Additionally, all staff members received 20 weekly newsletters by email during the first 6 months of the program. In terms of procedures, all team meetings started with discussing the practical assignment, followed by a presentation about the addressed skill, and a skills training including one or more interactive teaching methods. In the joint booster session, homecare staff practiced conversational skills in role-plays with professional actors. Team managers were also invited to the program meetings; they also received the weekly newsletters. An overview of the program is outlined in Fig. 2. A full description of the program, based on the Template for Intervention Description and Replication (TIDierR) checklist, has been published elsewhere [15]. The program applied in the current study differed slightly based on the learning from previous findings [18]. We added identifiable role models (program champions from the pilot study) to share their experiences with reablement, supporting materials to help homecare staff translate the program knowledge into practice (e.g., an exercise flyer, example communication questions and example goals and action plans), and a diploma ceremony for staff members who attended at least half of the program meetings.

### Implementation process

The program meetings were organized in the working areas in which the homecare staff was located. Two program trainers were present per meeting; they received a program manual and a 2-h training by one of the researchers before the program started, and short preparatory sessions before every program meeting. Homecare staff received an information letter and a program overview prior to the training, and presentation handouts, practical assignments and other supporting materials during every meeting. If staff members were unable to attend a meeting, we send them the materials by email. Additionally, we had regular contact with district nurses and team managers in-between the meetings to reflect on homecare staff's program engagement and, if applicable, to further tailor the program to staff's needs and wishes.

### Data collection

We collected both quantitative and qualitative data from homecare staff and program trainers to assess different research questions, in which the quantitative data provided a supportive role to the qualitative data [20, 24].

#### Background characteristics

Background characteristics (i.e., age, sex, educational level, job function, years of work experience and the number of hours worked weekly) were assessed through a baseline questionnaire during the first program meeting.

#### Process evaluation

We collected information on the process domains implementation, mechanisms of impact and context of the MRC framework [19], see Table 1. For implementation, we assessed the process indicators reach, dose, fidelity, adaptations and acceptability. All process domains and indicators are described below.

**Table 1:** Overview of process domains, indicators, definitions, and data collection

Process domains/ indicators	Data collection			
Definition	Data sources	Data collection methods	Operationalization	Timing of data collection
**Implementation**				
**Reach** Extent to which intended the audience comes into contact with program [19]	- Homecare staff	- Project logbook	- The number who refused, dropped out or completed the program - Reasons for refusals and dropouts	- Throughout the implementation
**Dose** Quantity of the program that was implemented[19]	- Homecare staff	- Registration forms	- The number who attended program meetings	- Prior to every program meeting
		-Checklists	- The number who conducted practical assignments	
		-Log data	- The number who consulted weekly newsletters	- Throughout the implementation
**Fidelity** Extent to which the program was implemented as planned[19]	- Researchers	- Project Logbook	- Performance according to the protocol	- Throughout the implementation
	- Homecare staff - Program trainers	- Focus group interviews	- Performance according to the protocol - Engagement in the program and with applying the program in practice	- After the implementation
**Adaptations** Alterations made to the program to achieve better contextual fit [19]	- Researchers	- Project Logbook	- If applicable: changes in content, procedures, activities and processes	- Throughout the implementation
**Acceptability** Extent to which participants were satisfied with the program[25]	- Homecare staff - Program trainers	- Focus group interviews	- Opinion about the program - Experiences with using the program in practice	- After the implementation
**Mechanisms of impact** Mechanisms through which the program activities may produce change [19]	- Homecare staff - Program trainers	- Focus group interviews	- Experienced changes in knowledge, attitude, skills, social and organizational support	- After the implementation
**Context** Factors external to the program that may influence the implementation [19]	- Homecare staff - Program trainers	- Focus group interviews	- Factors that may have facilitated or impeded the application of the program in practice	- After the implementation
	- Researchers	- Project Logbook	- Factors that may have facilitated or impeded the program’s implementation	- Throughout the implementation
**Suggestions for change**	- Homecare staff - Program trainers	- Focus group interviews	- Suggestions to improve the intervention or facilitate the implementation	- After the implementation

Note: adapted version of outcome measures of the process evaluation as published earlier [16]; client data and quantitative data on the mechanisms of impact will be covered in a separate article

*Reach* was defined as the extent to which the intended audience came into contact with the program. A project logbook was consulted to assess the number of staff members who refused, dropped-out or completed the program; reasons for refusals and dropouts were also assessed.

*Dose* was defined as the quantity of the program that was delivered by program trainers and received by homecare staff. Registration forms and checklists were completed prior to every program meeting to record homecare staff's number of program meetings attended and practical assignments completed. Log data from the software program LaPosta (LaPosta BV, Zutphen) monitored homecare staff's compliance towards consulting the weekly newsletters*.*

*Fidelity* was defined as the extent to which the program was implemented as planned. The project logbook was consulted to assess whether the program was conducted according to the protocol. *Adaptations* that were made to the program to achieve better contextual fit (i.e., changes in content, procedures, activities and processes) were also assessed. In addition, focus group interviews were conducted with homecare staff and program trainers after the implementation period of the program to gain insight into their degree of engagement in the program and with applying the program in practice. In total, five focus groups were performed: one with a subsample of nursing team members (November 2018), one with a subsample of domestic workers (November 2018), two with district nurses to interview all of them (December 2018), and one with the program trainers (August 2019). Subsamples were selected through quota sampling in a two-step selection process by one researcher (author THR). First, homecare staff who attended at least half of the program meetings were selected. Second, two nursing team members and two domestic workers per working area were invited by email, taking age, gender and years of work experience into account, to capture a wide range of perspectives [26]; when a staff member was unable or unwilling to participate, another staff member was invited. In total, four program trainers, six district nurses, ten nursing team members (i.e., two nurses, six nurse assistants and two nurse aides) and seven domestic workers participated in the interviews; they were all interviewed once. Author THR led the interviews with the homecare staff, assisted by one observer (author SFM or GARZ). Author GARZ led the interview with the program trainers. Interviews were performed at the healthcare organization and at Maastricht University, and were guided by pilot-tested interview guides that were developed for the current study based on the process domains and indicators (see Additional files 1 and 2). All interviews started with a 6-min video summarizing the program, were audio-recorded and lasted about 2 h. The main focus group findings were summarized at the end of each interview for participants to correct or add information.

*Acceptability* was defined as the extent to which homecare staff and program trainers were satisfied with the program. Their opinion about the program, including their most/ least appreciated program aspects, and experiences with using the program in practice were assessed by focus groups interviews as described above.

*Mechanisms of impact* were defined as mechanisms through which the program may produce change. Based on previous research, these mechanisms were assumed to be homecare staff's knowledge, attitude and skills about reablement, and social and organizational support from colleagues and team managers [6, 15, 17]. Therefore, by using the focus group interviews, information was collected about experienced changes in homecare staff's knowledge, attitude, skills, social and organizational support.

*Context* was defined as factors external to the program that may have influenced the program’s implementation. A project logbook was used to assess contextual factors. Additionally, the focus group interviews provided insight into factors that may have facilitated or impeded the application of the program in practice. In addition, *suggestions for change* were assessed to further improve the program or facilitate its implementation.

### Data analysis

Quantitative data were used to asses reach and dose, and were analyzed using descriptive statistics in SPSS Statistics for Windows version 25.0 (IBM Corp., Armonk, NY). Qualitative data were used to assess the remaining process domains and indicators, and were analyzed using a directed qualitative content approach in ATLAS.ti version 8.4 (Scientific Software Development GmbH, Berlin). Interviews were transcribed verbatim and coded using a coding scheme developed prior to the analysis based on the process domains and indicators. As the analysis proceeded, additional codes were generated by marking relevant sections, phrases or sentences. Two researchers (authors THR and RGMV) independently coded one-third of the transcripts. Author THR coded the remaining transcripts. Subsequently, the two researchers independently established categories by grouping codes. Any differences in coding or categorizing were discussed until consensus was reached. In a final phase, one researcher (author SFM) verified the categories and made minor adjustments in the allocation of the categories to the process domains and indicators in consultation with authors THR and RGMV. A detailed description of the findings supported with literal quotes from the focus group interviews, which were translated to English by a professional translator, are reported.

### Trustworthiness

Different strategies were adopted to ensure the trustworthiness of the findings regarding credibility, dependability, confirmability and transferability [27]. Credibility was ensured by prolonged engagement in the field, triangulation of data sources, data investigators (three researchers to code, analyze and interpret) and data collection methods. To ensure dependability and confirmability, four research partners (i.e., one nurse, one domestic worker, one older adult and one informal caregiver) were extensively involved in the research activities, from participating in the program meetings to commenting on the research findings. In addition, the procedures followed in this study were meticulously described. Transferability was ensured by describing the sample, setting and context in which the program was implemented.

## Results

### Implementation

#### Reach

The selected working areas included 67 nursing team members and 102 domestic workers, who delivered care to 354 clients. Most of them (*n* = 154) agreed to participate in the program (i.e., 23 nurses, 34 nurse assistants, 8 nurse aides and 89 domestic workers). Table 2 provides their baseline characteristics. Reasons for refusal included health problems and personal reasons. Some 140 staff members (90.9% of 154) were involved until the end of the program. The main reason for dropout was staff turnover.

**Table 2:** Baseline characteristics of homecare staff (*N* = 154)

Baseline characteristic	Nursing team members (*n* = 65)	Domestic workers (*n* = 89)
Age (years), mean (SD)	47.8 (12.4)	47.9 (10.7)
Sex (female), *n* (%)	62 (95.4)	88 (98.8)
Educational level, *n* (%)^a^		
Low	18 (27.7)	62 (69.7)
Intermediate	38 (58.5)	25 (28.1)
High	9 (13.8)	2 (2.2)
Job function, *n* (%)		
Registered nurse	23 (35.4)	–
Certified nurse assistant	34 (52.3)	–
Nurse aid	8 (12.3)	–
Work experience (years), mean (SD)	16.8 (12.3)	11.1 (8.3)
Working hours per week, mean (SD)	23.7 (6.4)	16.7 (5.5)

*n* sample size; *SD* standard deviation

^a^Low: Low vocational or advanced elementary education; Intermediate: Intermediate vocational or higher secondary education; High: Higher vocational education, university

#### Dose

All program meetings, practical assignments and 20 weekly newsletters were delivered, with the exception of two newsletters that were sent to only 80 and 90% of the staff members due to technical issues. On average, nursing team members and domestic workers attended 66.6 and 78.4% of the program meetings, respectively. Nevertheless, compared to the kick-off meeting, all the following meetings were less well attended (Table 3). The majority of nursing team members (73.8%) and domestic workers (86.5%) attended at least half of the meetings and received a diploma. Eight nursing team members (12.3%) and 39 domestic workers (43.8%) attended all meetings. Main reasons for not attending meetings were illness or vacation. Additionally, on average, nursing team members and domestic workers conducted 55.4 and 57.6% of the practical assignments and consulted 76.5 and 42.1% of the weekly newsletters, respectively.

**Table 3:** Dose delivered, dose received and number of homecare staff members invited and present per meeting (*N* = 154)

	Nursing team members (*n* = 65)		Domestic workers (*n* = 89)	
Program components	Dose delivered	Average dose received (%)	Dose delivered	Average dose received (%)
Program meetings	7	4.7 (66.6)	5	3.9 (78.4)
Practical assignments	6	3.3 (55.4)	4	2.3 (57.6)
Weekly newsletters ^a^	20	15.3 (76.5)	20	8.4 (42.1)
Diploma ^b^	–	48 (73.8)	–	77 (86.5)
**Program meetings**	*n* invited ^c^	*n* present (%)	*n* invited ^c^	*n* present (%)
Kick-off meeting	65	55 (84.6)	89	77 (86.5)
Team meeting 1	65	43 (66.2)	88	75 (85.2)
Team meeting 2	63	43 (68.3)	–	–
Team meeting 3	62	38 (61.3)	87	72 (82.8)
Team meeting 4	62	41 (66.1)	–	–
Team meeting 5	60	46 (76.8)	84	67 (75.3)
Booster session	57	37 (64.9)	83	58 (65.2)

*n* sample size

^a^All 20 newsletters were sent, with the exception of two that were only sent to 80 and 90% of all professionals, respectively, due to technical issues

^b^Staff members who attended at least half of the program meetings received a diploma (i.e., four meetings for nurses and three for domestic workers)

^c^Fewer people were invited per meeting as the program progressed due to dropout

The following process domains and indicators were mainly analyzed using the focus group data. The educational level of the staff members interviewed was significantly higher than that of the remaining homecare staff who participated in the program (78.3 and 42.7% had an intermediate or high educational level, respectively).

#### Fidelity

The project logbook showed no major deviations from the protocol. Program trainers felt sufficiently prepared to provide the program meetings and covered all components of the program meetings (i.e., discussing the practical assignment, skills presentation, and interactive teaching methods). Only the time spent on the different components varied due to the teams’ different needs; nevertheless, the skills presentation often took up most of the time.

According to the homecare staff and program trainers interviewed, there was variation in the extent to which staff members engaged during program meetings. The program trainers assumed that the large training groups, on average 14.3 ± 5.1 staff members at team meetings and 25.5 ± 9.1 at the kick-off/ booster session, undermined homecare staff's active participation. Furthermore, they surmised that staff members did not always actively participate because the meetings predominantly focused on explaining rather than practicing skills.

*We frequently used PowerPoint presentations (during the program), and people may or may not be learning from those. In the healthcare organization, on the other hand, we would normally use more interactive methods. I think that is a little more effective. (program trainer 1).*

#### Adaptations

One minor adaptation was made to the program’s implementation. Due to variation in homecare staff's program engagement, the district nurses and team managers were asked to emphasize the importance of participating in the program to their teams by mail, or during team meetings not related to the program. No changes were made to the program itself. Nevertheless, program trainers were able to share more examples from practice after the first wave of trainings due to the shared experiences during this wave.

#### Acceptability

Staff members were generally satisfied with the program. They particularly valued the program’s practical elements (i.e., role-plays, booklet with practice exercises, weekly newsletters). According to many staff members, the role-plays provided insight into how to encourage clients in practice and helped to reflect on their own behavior. Some domestic workers felt out of their comfort zone, though, as the professional actors always remained in their role, challenging them to react verbally and behaviorally.

*They (the actors) stayed in their role while I was thinking about how to respond. That was pretty intense. But I did get a taste of what it would be like in real life, and I also learned from the way others responded. (domestic worker 4).*

Most nursing team members also appreciated the booklet with practice exercises, which contained comprehensible examples of how to remain active at an older age. This supported them to motivate clients to participate in daily and physical activities.

*(I liked) the booklet with the practice exercises, because if I am telling clients that they should be keeping physically active, now at least I can show them what kind of exercises will help them. (nurse assistant 4)*

According to some staff members, the practical assignments in general helped to practice skills in an accessible manner, and to reflect on one’s own actions so as to engage in a process of continuous learning. Others, though, considered the assignments a burden, due to an experienced lack of time or because they were not used to putting things down on paper.

Many staff members also considered that the newsletters were useful reminders with valuable information about the benefits of remaining physically active and practical tips on how to motivate clients towards performing activities for themselves. This supported staff members in conversations with clients about stimulating independence.

*I especially liked the newsletters with the tips, which also provide some explanation and background information. Some of our team members even showed the newsletters to their people (clients). It helped them to explain to clients why it is good to stay physically active, because it is quite difficult to explain something like that. (district nurse 3).*

Some district nurses, though, felt that their colleagues read fewer newsletters towards the end of the program due to their high frequency (once per week).

Most staff members also appreciated the program’s team approach, as this allowed them to get to know each other and to exchange experiences about what clients can still do themselves and how to approach them in the best way. Many staff members indicated that they would like to interact even more with colleagues both during and outside the program, so as to learn from each other’s working practices.

*There now is a lot of interaction (with colleagues), and that encourages people to ask each other questions, like: ‘Oh I saw you with that client, so how would you approach this?’. (nurse assistant 5).*

Staff members' opinions were divided regarding whether the program fitted with their daily practice. District nurses and program trainers indicated that the program fitted the roles and tasks of staff members; however, some district nurses considered the meeting on goal setting and action planning possibly too difficult for nurse assistants and nurse aides. Consequently, one district nurse did the assignment on goal setting and action planning together with the team to support them.

*Everyone had to set goals and design action plans for their own clients. I noticed that most of the nurses could do that quite well, but some of the others found it harder (nurse assistants and nurse aides). They were not sure how to describe some of the things they do. They know what they are doing in practice, but they do not always know how to describe that properly. (district nurse 3).*

Many nursing team members indicated that they were already familiar with most of the program content due to a previous short training on self-management.

Opinions were also divided regarding the program’s duration. Most staff members appreciated the program’s gradual structure, allowing them to implement changes step-by-step by alternating between learning, experimenting, and reflecting. Some nursing team members, though, considered the team meetings of 1 h too short to practice skills and some domestic workers considered the 2 months in-between the team meetings too long to remain continuously aware of the program while providing homecare services.

### Mechanisms of impact

#### Experienced changes in homecare staff's knowledge

According to many staff members, their knowledge regarding reablement improved because of the program; they mentioned several benefits for older adults (e.g., more confidence in performing activities) and for themselves (e.g., increased work efficiency). They also mentioned tips on alternative ways of providing care (e.g., using an eyedropper or grabber) and other strategies to improve clients’ activity levels (e.g., deploying volunteers for doing groceries together). This knowledge raised awareness among staff members, which helped to change their way of thinking towards encouraging independence among homecare clients.

*If there is a client who needs to be showered twice a week, then you would also help them to get dressed afterwards. But for the rest of the week, they would be doing that by themselves, so I do not actually need to help them to do that. So sometimes it is just about thinking differently. (nurse assistant 4).*

The program trainers, though, found that some staff members considered certain parts of the program as complex (e.g., implementing goalsetting and action planning); they therefore expected that not all program content led to increased knowledge of staff members. Most district nurses, on the other hand, indicated that the knowledge to encourage clients towards independence was generally present, but that homecare staff sometimes considered it challenging to integrate this knowledge into their working practices.

*In the past, the work was really about taking care of people and basically doing everything for them. That is how I learned to do it from the start. And that makes it hard to think about your work in a different way. (nurse aide 1).*

#### Experienced changes in homecare staff's attitude

Most staff members experienced positive changes in their attitude towards reablement due to the program. Reasons frequently mentioned for this were successes that they gained regarding encouraging clients to perform activities themselves again, and the impact that this had on clients’ view about themselves.

*When I go to visit a client, and I find them standing by the door waiting to tell me that they have cleaned out their cupboard on their own – that is what makes it really worthwhile! They look so proud of themselves and that is motivating because we have really achieved something. (domestic worker 6).*

*He feels more involved now. Before, he used to think he could not do anything for himself anymore, but now he is happy that he is able to do things independently. He does not have to bother other people anymore. (domestic worker 1).*

Some staff members were still doubtful about reablement, though, for instance, due to negative outcome expectations of reablement (e.g., implementing reablement takes more time and staff members are now being left with only the more challenging tasks), a preference for or habit of taking over activities, or a short-term vision of care where it is faster to take over tasks.

#### Experienced changes in homecare staff's skills

*Team meetings 1 and 2:* Many staff members now used the different communication strategies that were part of the program more consciously in practice, such as motivating and complimenting clients, being firm, negotiating and joking. They also shared more tips with clients on how to engage in daily and physical activities.

*Now I talk more to the clients about their health - and for example, when I tell them about the 10% decrease in muscle mass, that really gets them thinking. I had never thought about mentioning that, but it really opens their eyes. (domestic worker 6).*

The program trainers, however, indicated that homecare staff's conversational skills varied considerably; they therefore assumed that conversations might not always have been conducted in the correct manner.

*Team meeting 3:* Most district nurses spent more attention on reablement during the needs assessment with newly referred clients because of the program. They also formulated client-centered goals more specifically to clarify to their colleagues which activities (not) to take over.

*I learned to set more specific goals. In the past, I would often formulate goals like ‘performing ADL’, or ‘showering or washing at the washbasin’, but things are not always clear then. It does not say, for instance, that he (the client) should try to wash his upper body himself. (district nurse 4).*

Most domestic workers often did not work with goals and action plans, but sometimes made verbal agreements with clients.

*Team meeting 4:* Many district nurses spoke more frequently with the social network of clients than before the program, for instance, about clients’ resistance to change. Some other nursing team members also had more contact with the social network.

*We had one lady who was slowly able to start doing more things for herself again, but her son used to stop her all the time. He would say: ‘(Staff member X) will be here soon, so leave that for her to do.’ I told him that if she can do things for herself, she really should be doing them because that is much better for her. (nurse assistant 3).*

Some district nurses, however, indicated that not everyone was equipped for such conversations, especially when the social network unnecessarily took over activities and resisted the change. The domestic workers rarely had substantive contact with the social network of clients.

*Team meeting 5:* Many staff members assessed clients’ capabilities more often because of the program; they looked more consciously at what clients could still do themselves, and better defined to clients what they could expect from them.

*In the past, when I had a new client, I would just arrive at the agreed time and start working right away. But now I arrive 10 to 15 min early and I use that time to talk (to the client) about what they are still able to do and what I can do for them. Then they know what they can expect from me and what I will expect from them, and I can refer back to that. (domestic worker 4).*

The district nurses, though, indicated that the extent to which homecare staff succeeded in this varied. They also got the impression that some staff members considered the assessment of capabilities the responsibility of the district nurse; they therefore expected that staff members did not always consciously look at what clients could still do themselves.

#### Experienced changes in homecare staff's social and organizational support

Most district nurses indicated that the program led to greater support and cooperation within the team. They spoke more frequently with their team about how to implement reablement in practice; they also set goals more often in consultation with the team to create a shared responsibility in the care provided. Consequently, most nursing team members started to report more extensively on these goals, thereby gaining more insight into each other’s working routines. If someone on the team deviated from agreements made in the team, they were called to account by colleagues, thereby reducing differences in their working practices. Most domestic workers had little contact with their colleagues outside the program, although some did have more contact with nursing team members than before.

*In the past, we would just do the household chores, and the nurses would get on with their own work. But since the program, we have started talking more. Now it feels more like a joint effort. (domestic worker 7).*

According to the staff members interviewed, the extent to which team managers attended program meetings and supported homecare staff towards implementing reablement in practice, varied. Most domestic workers and some district nurses valued the presence and support of team managers during program meetings and meetings not related to the program.

*My team found it (the support of the team manager) very positive. The interest, the compliments, and the personal attention - they appreciated that. (district nurse 1).*

Many staff members also felt free to consult the team manager in challenging situations. Nevertheless, some domestic workers considered it challenging to express themselves when the team manager was present at the program meetings or to approach the team manager when they encountered problems.

### Context

#### Contextual facilitators

Homecare staff mentioned several contextual factors that facilitated the program’s implementation. First, the use of digital care plans provided nursing team members with better insight into goals that were set with clients, which made them more inclined to report on them. Besides, care plans were accessible to the clients’ social network, allowing them to monitor the care process (i.e., the new way of working). Second, the healthcare organization’s lump sum funding system (meaning that the organization receives a fixed amount of money per client irrespective of the amount of care delivered) ensured that staff members were less time-bound when providing care. Lastly, newly referred clients could be more easily encouraged, since they did not experience the old system of care provision in which activities were often taken over.

*What I see is that our team still makes the most progress with new clients who need care. They (the staff) actively focus on engaging them (newly referred clients). (district nurse 4)*

#### Contextual barriers

Homecare staff also mentioned some contextual barriers. First, resistance to change from clients or their social network impeded staff members in implementing reablement. Reasons frequently mentioned for this were older adults feeling too old or too weak, feeling entitled to care, being afraid of losing care, or having unrealistic expectations about homecare that were created by other stakeholders (e.g., hospitals, general practitioners). Second, nursing team members still struggled to encourage clients in complex care situations. Lastly, particularly nursing team members indicated that time pressure and staff shortages could lead to taking over activities.

*Time pressure - for me that is one of the biggest challenges. You have a busy day, and you know that people need to receive care at a particular time, so very quickly you’re inclined to just say ‘let me do that for you’ (the clients). (nurse aide 2).*

### Suggestions for change

Homecare staff and program trainers had some suggestions to improve the program and facilitate its practice implementation. To improve homecare staff's attendance rates and program engagement, staff members suggested to make the training mandatory and program trainers suggested smaller training groups and more interactive teaching methods. To improve homecare staff's knowledge, attitude, skills, social and organizational support, staff members suggested to include training on-the-job, practical assignments on a team level and more role-plays. Program trainers suggested to further tailor the program to homecare staff's needs and wishes to better support them during the process of behavior change. Additionally, they suggested to first train team managers and district nurses about how to support staff in implementing reablement before training staff members. To reduce resistance to change, homecare staff and program trainers suggested providing information on reablement to clients, their social network, and to other relevant stakeholders.

## Discussion

This comprehensive process evaluation provided insight into the implementation, mechanisms of impact and context of the reablement training program ‘Stay Active at Home’ program that was implemented in the daily practice of Dutch homecare for older adults. The program was largely implemented as intended. Homecare staff's compliance with attending the program meetings was reasonably good; however, the extent to which staff members conducted the practical assignments and consulted the weekly newsletters varied. They experienced positive changes in their knowledge about and attitude towards reablement, learned new skills or further developed already existing skills to encourage clients towards independence, and perceived social and organizational support from colleagues and team managers to implement reablement in practice. Contextual factors that potentially facilitated the implementation were digital care plans, the organizations’ funding model (lump sum funding instead of fee-per-hour) and newly referred clients. Potential barriers were resistance to change, complex care situations, and time pressure and staff shortages.

To understand how the program may have produced a change in homecare staff's behavior, it is essential to unravel its mechanisms of impact [19]. Based on previous research, homecare staff's knowledge, attitude, skills, and perceived support are assumed to contribute to the desired behavior change [6, 17]. A possible precondition for bringing about change in these behavioral determinants is intervention acceptability. Homecare staff mainly valued the program’s practical elements (i.e., role-plays, booklet with practice exercises, newsletters) and team approach. Role-plays provided a realistic representation of homecare staff's behavior, which helped to reflect on their own skills and on the extent to which they applied these in practice. Since learning through observation and reflection can be quite useful when working towards behavior change, this may have facilitated homecare staff to integrate reablement into their working practices [28]. The booklet with practice exercises and newsletters provided comprehensible examples and practical tips for remaining active in daily life; this encouraged homecare staff to discuss with clients the importance of performing activities themselves. These tools may therefore have eased the translation from the program knowledge into practice [29]. The team approach allowed homecare staff to exchange practice experiences, work together on goals, and improve their conversational and collaboration skills. This may also have facilitated the intended behavior change, as regular team support and a framework for cooperating and applying professional expertise and judgment are assumed essential in the adoption of reablement [30, 31].

Although most staff members held a positive opinion about reablement, it was sometimes difficult to change their behavior. This is in line with other studies confirming that working with reablement can be challenging [7, 9, 10]. Behavior change is a complex process with various prerequisites, such as being receptive to new ideas, understanding the desired behavior, willingness to change, and being able to change [32]. A lack of *receptiveness* may have influenced staff members' program compliance. Domestic workers were more compliant with attending the program meetings than nursing team members, possibly because they receive little training in the Netherlands, thereby making them more eager to participate. Another explanation may be that mainly nursing team members suffered from time restraints and staff shortages. A lack of *understanding* may have limited staff members in internalizing reablement. Some program parts were experienced as difficult and some found it challenging to integrate the program knowledge into practice, which is in line with other research [33]; this implies that the knowledge transfer and homecare staff's understanding of reablement was not optimal. This may be explained by variation in staff members' educational level and experience, so the program may not always fitted their prior knowledge. Staff members' *willingness* to adopt reablement may have been impeded by a preference for or habit of taking over activities or by negative outcome expectations of reablement. This indicates that staff members were likely in different stages of behavior change and that the program possibly did not always meet their needs and wishes to successfully move to the next stage [30, 32]. Staff members' *ability* to change may have been impeded by a variety of other behavioral factors (e.g., lack of confidence), contextual factors (e.g., unclear roles, responsibilities or expectations), or by a combination thereof. Nevertheless, by implementing ‘Stay Active at Home’ in Dutch homecare practice, we have been able to take the first steps in changing homecare staff's behavior from taking over activities of older adults towards encouraging older adults to perform activities for themselves.

Future program implementation may benefit from minor adaptations. First, further tailoring the program to homecare staff's needs and wishes may likely improve their engagement in the program and with delivering the program in daily practice. In doing so, it might be valuable to take into account differences in educational backgrounds and experiences, so that the program is explained in a comprehensible manner to all staff members involved [34]. In addition, taking into consideration the different roles and responsibilities of staff members in providing care could possibly contribute to them feeling more responsible for the implementation of reablement [30, 34]. Second, implementing reablement requires patience and time from staff members. This implies that healthcare systems and policy-makers should support a stimulating work environment, thereby considering the extra efforts and time needed to change homecare staff's work practices. One must keep in mind, though, that reablement is a relatively new approach, so its evidence base is still limited and inconsistent [7, 12, 13, 17, 35]. Although the current evidence suggests that reablement is a promising approach, further research on the ‘Stay Active at Home’ program in terms of its (cost-) effectiveness for homecare clients and healthcare systems is needed prior to a broader implementation [7].

This study has several strengths. Using the MRC framework provided a profound understanding of the program and its implementation, and gave indications for mechanisms of impact and contextual factors that may influence the intended behavior change [19]. Furthermore, combining multiple qualitative and quantitative methods, incorporating data from homecare staff and program trainers, using three researchers to analyze the data, and collaborating with relevant stakeholders, increased the trustworthiness of the findings [27]. Limitations of the study included the higher educational level of the staff members interviewed compared to the remaining homecare staff who participated in the program, due to an overrepresentation of district nurses. Besides, motivated homecare staff may have been overrepresented in the interviews because only those who had reasonable program exposure (i.e., attendance of at least half of the program meetings) were selected. Nevertheless, the more interviews were conducted, the fewer new codes were generated, which may indicate data saturation. A second limitation may be social desirability bias of homecare staff because the researcher who performed the interviews with the staff members was also involved as program trainer. Moreover, two of the program trainers were researchers, thereby potentially introducing experimenter bias. We tried to minimize bias by using a pilot-tested interview guide, indicating to interviewees that data would be pseudonymized and treated with confidence, and appointing a moderator who was not involved in the program for the interviews with program trainers. Third, the staff members interviewed were predominantly positive about using the program in practice, while program trainers were more critical towards homecare staff’ practice performance. Video- or audiotaping would have been valuable to add to the data collection methods to provide more valid information about homecare staff's actual performance in practice [36].

## Conclusions

The reablement training program ‘Stay Active at Home’ program was feasible to implement in Dutch homecare and was perceived as useful in daily practice. The program seemed to have a positive impact on homecare staff's knowledge, attitude, skills, social and organizational support to implement reablement. However, integrating reablement in homecare staff's working practices remained challenging due to personal and contextual factors. This study contributes to the growing body of evidence that shifting homecare services from ‘doing for’ towards ‘doing with’ older adults involves a major paradigm shift for homecare staff. Future program implementation may benefit from minor program adaptations and a more stimulating work environment.

## Supplementary Information

**Additional file 1. **Interview guide for focus group interviews with homecare staff (*n* = 23).

**Additional file 2. **Interview guide for focus group interview with program trainers (*n* = 4).

## Data Availability

The datasets generated and/or analyzed during the current study are available from the corresponding author on reasonable request.

## Abbreviations

cRCT: Cluster Randomized Controlled Trial
(I)ADLs: (Instrumental) activities of daily living
MRC: Medical Research Council
