# Supplementary material for: Process evaluation of a reablement training program for homecare staff to encourage independence in community-dwelling older adults
Source: BMC Geriatr. 2021 Jan 6;21:5. doi: 10.1186/s12877-020-01936-7 (PMC7789187; doi:10.1186/s12877-020-01936-7)
Supplement: Supplementary file 1 — Additional file 1. Interview guide for focus group interviews with homecare staff (n = 23). [file 12877_2020_1936_MOESM1_ESM.docx]

| **Supplementary file 1.** Interview guide for focus group interviews with homecare staff (n = 23). | |
| --- | --- |
| Process domains/indicators and interview questions | |
| **Implementation** | |
|  | - To what extent did you actively engage during program meetings? (fidelity) |
|  | - To what extent did you apply the program in practice? (fidelity) |
|  | - What did you think of the program in general (e.g., program rationale, content, teaching methods and duration)? (acceptability) |
|  | - What program aspects did you appreciate the most/ least (e.g., program meetings, practical assignments, weekly newsletters)? Why? (acceptability) |
|  | - To what extent did the program fit into your daily practice? (acceptability) |
| **Mechanisms of impact** | |
|  | - What did you learn from the program? (knowledge, skills) |
|  | - To what extent did the program influence your way of thinking/ working? (attitude) |
|  | - To what extent did you receive social and organizational support from colleagues and the organization to apply the program in practice? (social and organizational support) |
|  | - What experiences did you have with applying the program in practice? |
| **Context** | |
|  | - What contextual factors facilitated/impeded you in attending the program meetings? |
|  | - What contextual factors facilitated/ impeded you in applying the program in practice? |
| **Suggestions for change** | |
|  | - Do you have suggestions to improve the program (e.g., planning, program content, teaching methods and duration)? |
|  | - Do you have suggestions to facilitate the implementation of the program in practice? |
|  | - What do you need to continue to apply the program in practice? |
