# Supplementary material for: Process evaluation of a reablement training program for homecare staff to encourage independence in community-dwelling older adults
Source: BMC Geriatr. 2021 Jan 6;21:5. doi: 10.1186/s12877-020-01936-7 (PMC7789187; doi:10.1186/s12877-020-01936-7)
Supplement: Supplementary file 2 — Additional file 2. Interview guide for focus group interview with program trainers (n = 4). [file 12877_2020_1936_MOESM2_ESM.docx]

| **Supplementary file 2.** Interview guide for focus group interview with program trainers (n = 4). | |
| --- | --- |
| Process domains/indicators and interview questions | |
| **Implementation** | |
|  | - To what extent did you feel facilitated and prepared to provide the program meetings? (fidelity) |
|  | - How would you reflect on your own functioning in providing the program meetings? (fidelity) |
|  | - To what extent did homecare providers actively engage during program meetings? (fidelity) |
|  | - To what extent did you think homecare providers applied the program in practice? (fidelity) |
|  | - What did you think of the program in general (e.g., program rationale, content, teaching methods and duration)? (acceptability) |
|  | - To what extent did the program fit in with other programs offered by the healthcare organization? (acceptability) |
| **Mechanisms of impact** | |
|  | - To what extent did you think the program influenced homecare providers' knowledge, attitude and skills? |
|  | - To what extent did you think homecare providers received social and organizational support from colleagues and the organization to apply the program in practice? |
| **Context** | |
|  | - What contextual factors facilitated/impeded you in providing the program meetings? |
|  | - What contextual factors may have facilitated/ impeded homecare providers in attending the program meetings? |
|  | - What contextual factors may have facilitated/ impeded homecare providers in applying the program in practice? |
| **Suggestions for change** | |
|  | - Do you have suggestions to improve the program (e.g., planning, program content, teaching methods and duration)? |
|  | - Do you have suggestions to facilitate the implementation of the program in practice? |
